# The Utility of Percutaneous Balloon Kyphoplasty for Treatment of Traumatic Vertebral Compression Fracture to Prevent Opioid Dependence in a Young Opioid-Dependent Patient

**DOI:** 10.7759/cureus.9733

**Published:** 2020-08-14

**Authors:** Hisham Kassem, Ivan Urits, Samara Shipon, Jamal Hasoon, Omar Viswanath

**Affiliations:** 1 Anesthesiology and Perioperative Medicine, Mount Sinai Medical Center, Miami Beach, USA; 2 Anesthesiology, Critical Care, and Pain Medicine, Beth Israel Deaconess Medical Center and Harvard Medical School, Boston, USA; 3 Pain Management, Valley Pain Consultants - Envision Physician Services, Phoenix, USA

**Keywords:** verterbal compression fracture, kyphoplasty, opioid-sparing analgesia

## Abstract

Vertebral compression fractures are often found in the elderly population with known risk factors. Less commonly, they may occur in otherwise healthy patients following traumatic falls and can cause significant pain requiring opioid therapy. This case emphasizes the use of percutaneous balloon kyphoplasty as an effective treatment strategy in a young opioid-dependent patient as a means to support the return to baseline functionality.

## Introduction

Vertebral compression fractures are most commonly seen in the elderly population or in patients with specific risk factors including osteoporosis, chronic steroid usage, and osseous neoplastic lesions among others [1]. The condition may be associated with significant pain, impairing overall quality of life and functional ability. Patients may often be treated with conservative management such as rest, bracing, medications, minimally invasive intervention, or surgical intervention [1]. The use of percutaneous balloon kyphoplasty has emerged as a safe and effective intervention to assist in providing pain relief [2]. Although far less common, traumatic vertebral compression fractures can occur in the younger patient population with significant impact to their functionality, yet there remains to be a current consensus on the ideal treatment strategy.

## Case presentation

**Figure 1:**
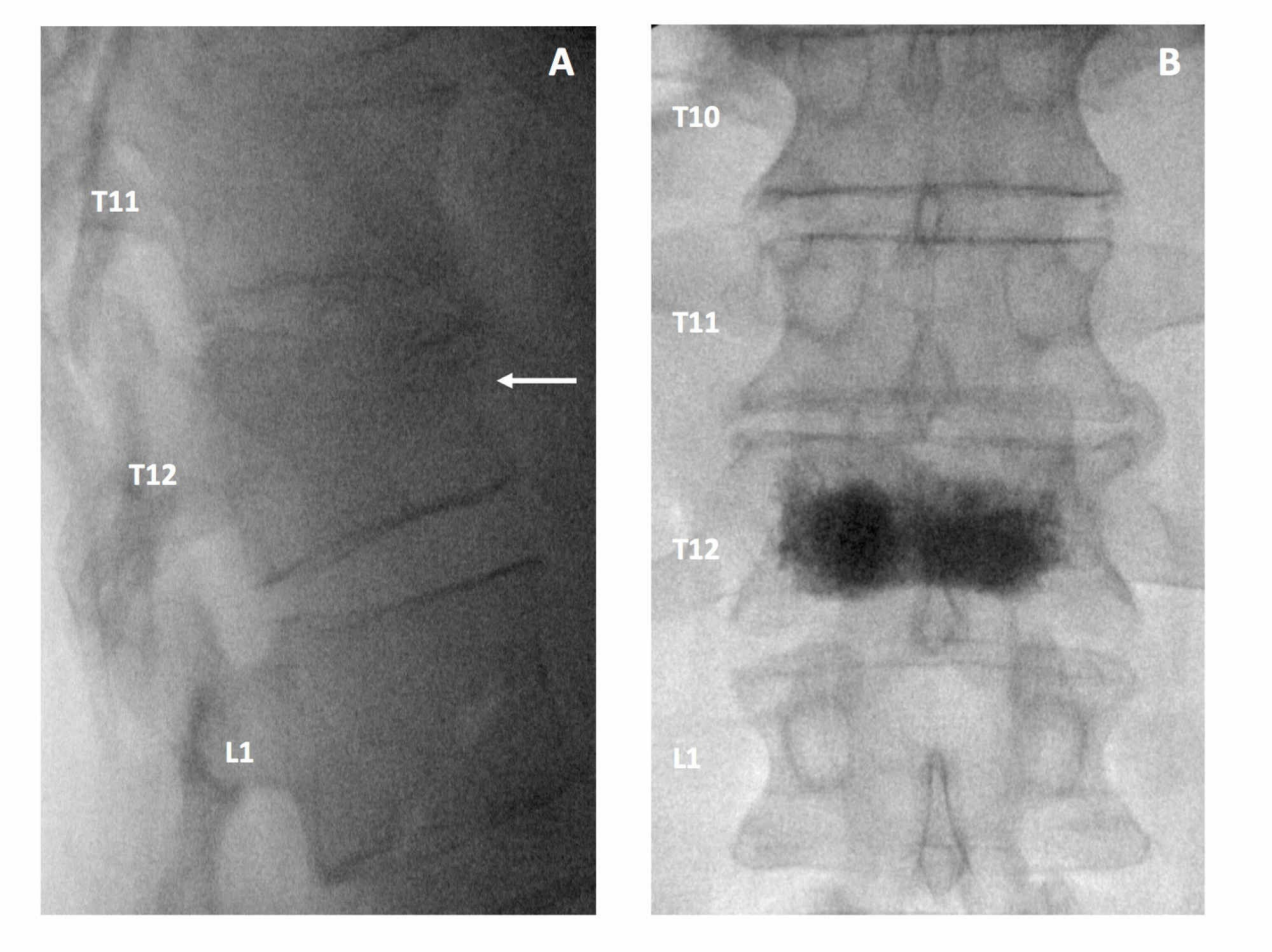
Intraoperative Fluoroscopy Figure 1A: Lateral fluoroscopic view of thoracolumbar spine demonstrating vertebral compression fracture at T12 with anterior wedging and loss of height Figure 1B: Intraoperative anteroposterior (AP) fluoroscopy demonstrating successful cement augmentation of the T12 vertebral body without extravasation of contrast

A 38-year-old otherwise healthy male presented with a chief complaint of axial lower thoracic back, which began following a fall from the standing position while roller skating with his child. Magnetic resonance imaging (MRI) of the thoracolumbar spine demonstrated a vertebral compression fracture at T12 with 35% height loss and associated edema. The patient had returned to work with the use of oxycodone three times per day as needed for approximately three months. However, he complained of side effects with these medications - suboptimal pain relief, as well as the inability to have a quality of life after his work. As such, he presented for consideration of alternative treatment options, specifically consideration of kyphoplasty. Repeat imaging was obtained and showed stable height loss; however, there was persistent edema on short-tau inversion-recovery (STIR) and T1 weighted imaging. Since the patient continued to have severe and functionally limiting pain greater than 8 out of 10 with affected activities of daily living and created dependency on opioid medications with side effects, the decision to proceed with percutaneous balloon kyphoplasty was made. This was performed at the T12 vertebral level with the successful restoration of vertebral body height and adequate placement of contrast-enhanced cement confirmed with intraoperative fluoroscopy (Figures 1A-1B). At one week follow up, the patient noted complete resolution of his pain and increased functional ability. At six weeks post-procedure follow up, the patient continued to do well and most importantly, had stopped the use of all opioid medications, and was able to enjoy a quality of life with his family.

## Discussion

This clinical scenario is an example of using percutaneous balloon kyphoplasty as an applicable method to reduce pain, lessen short and long term debility, reduce opioid dependency, and improve quality of life even in a young patient, who is not representative of the typical patient population. A study by Hirsch et al. concluded that vertebral augmentation presented a prominent mortality benefit over nonsurgical management for vertebral compression fractures, evidenced by a low number needed to treat to save one life at one year and up to five years after vertebral augmentation [3]. This suggests that as the patient population can be expanded to include younger patients, there is an inherent benefit from a low number requiring treatment. The possibility of significant thoracic kyphosis can be seen if these fractures are allowed to heal without intervention affecting a patient’s pulmonary function and compromising a young patient's active lifestyle [4]. Moreover, the pain may require pain control with opioids, which carry their own inherent risk for use and misuse. There has been a noted benefit to earlier treatment with vertebral augmentation as this is a predictor of persistent opioid use post procedure, especially in opioid-naive patients [5]. While this procedure is relatively safe and effective, it is important to acknowledge it is not without potential risks and adverse events. These risks include infection, bleeding, allergic reactions to bone cement, and the possibility of a pulmonary embolism, among others [2]. This case strengthens the notion that vertebral stabilization is a practical option even for younger patients with vertebral compression fractures not secondary to osteoporosis or malignancy, but further large prospective studies may elucidate the utility of percutaneous balloon kyphoplasty in the setting of acute traumatic vertebral compression fractures especially in young, opioid-dependent patients.

## Conclusions

While this case presents anecdotal evidence supporting the benefit of vertebral augmentation for a specific patient population, we believe there is a wide potential to offer this nonsurgical treatment as an alternative to opioids. There is a perceived benefit based on recent literature for the use of percutaneous balloon kyphoplasty in an outpatient setting.
